# Adenoma miss rate determined by very shortly repeated colonoscopy: Retrospective analysis of data from a single tertiary medical center in China

**DOI:** 10.1097/MD.0000000000012297

**Published:** 2018-09-21

**Authors:** Cheng-Long Wang, Zhi-Ping Huang, Kai Chen, Fei-Hu Yan, Liang-Liang Zhu, Yong-Qi Shan, Yong-Jun-Yi Gao, Bai-Rong Li, Hao Wang, En-Da Yu, Zi-Ye Zhao

**Affiliations:** a Department of Colorectal Surgery and GI Endoscopy Center, Changhai Hospital; b Department of General Surgery, Guangzhou General Hospital of PLA, Guangzhou; c Department of General Surgery, Shenyang General Hospital of PLA, Shenyang; d Department of Emergency Medicine, The 309th Hospital of PLA, Beijing; e Department of Gastroenterology, Changhai Hospital, Naval Medical University, Shanghai, China.

**Keywords:** adenoma miss rate (AMR), colonoscopy, quality control, tertiary hospital

## Abstract

Adenoma miss rate (AMR) has been calculated in several tandem colonoscopy studies, but it costs overmuch to carry out a clinical trial.

We aimed to put forward AMR by taking advantage of retrospective data, and to judge the comparability between AMRs from prospective and retrospective data.

Data of the patients accepting repeated colonoscopies during January to September 2016 was retrospectively collected and analyzed. Information was recorded, including bowel preparation quality of the first colonoscopy, size, location, histology and whether missed within the first colonoscopy of each single adenoma. AMR was compared by different risk factors through *χ*^2^ test and multivariable logistic regression.

Around 267 adenomas were detected during 309 pairs of repeated colonoscopies, of which 66 were missed during the first colonoscopies. AMRs of the lesions small in size, nonadvanced in histology, in poor bowel preparation context and located in the proximal colon, were significantly higher than the opposite ones, and old age and male were related to adenoma missing (*P < *.05). In multivariable logistic regression analysis, adenoma-related factors (diminutive in size, poor bowel preparation and located in ascending colon, transverse colon or sigmoid colon), and patient-related factors (older than 60 years, male and poor bowel preparation) were found to be independently associated with missing adenomas (*P < *.05).

AMR of retrospective data is comparable to that of tandem studies. Several risk factors influence AMR dramatically, which should be paid attention to.

## 1 Introduction

Detection and removal of adenomas by colonoscopy is proven effective in reducing the incidence of colorectal cancer (CRC), and colonoscopy is widely recognized as the gold standard for CRC screening.^[1,2]^ However, missing adenomas during colonoscopy has been well documented and the missed ones actually cause interval CRCs, which compromise the effectiveness of colonoscopy. Researchers have found adenoma detection rate (ADR) is closely associated with interval CRC, and ADR is now regarded as the core index of colonoscopy quality.^[3]^

In order to acquire detail information about missed adenomas and to supplement ADR, adenoma miss rate (AMR) has been brought out. To calculate AMR, tandem colonoscopies, which means 2 colonoscopies with polypectomy taken in the same patient within very short time, must be carried out. According to this examination, AMR refers the proportion of adenomas missed by the first colonoscopy to total adenomas. Former researches reported an AMR of 20% to 47.9%,^[4–7]^ and a systematic review gave the pooled AMR of 22% in 2006.^[8]^ However, to carry out tandem exam is time and money-consuming, and it is not appropriate to perform tandem exam in clinical practice due to medical ethic issues, so its utility is largely limited. Till now, very limited data of miss rate have been reported and most of them are from North America and European countries. It is not clear how is the situation in China, where morbidity of CRC is increasing dramatically.^[9]^

Here we report AMR in China by analyzing the data of shortly repeated colonoscopies in a tertiary gastrointestinal (GI) endoscopy center. To our knowledge, this is the first report of AMR in the context of routine clinical practice in China, so that we are able to have an idea about how is our colonoscopy quality.

## 2 Materials and methods

### 2.1 Study design

This study was undertaken in a tertiary medical center GI endoscopy unit (Changhai Hospital, Naval Medical University, Shanghai, China). All patients were referred from outpatient and inpatient of the same medical center. They all completed a standardized tick box questionnaire on main complain and lower GI symptoms, so the indications for colonoscopy were recorded and available for latter research. All the information about patients’ age, gender, indications, images of endoscopic examinations, and endoscopic findings were all recorded in an endoscopic database (Endoscopy Information System, Angelwin, Beijing, China) prospectively, after obtaining informed consent. Together with pathological information from the department of pathology, the information we needed was ready for referring.

This study was approved by the Medical Ethics Committee, Changhai Hospital, Naval Medical University.

### 2.2 Study population

**Figure 1:**
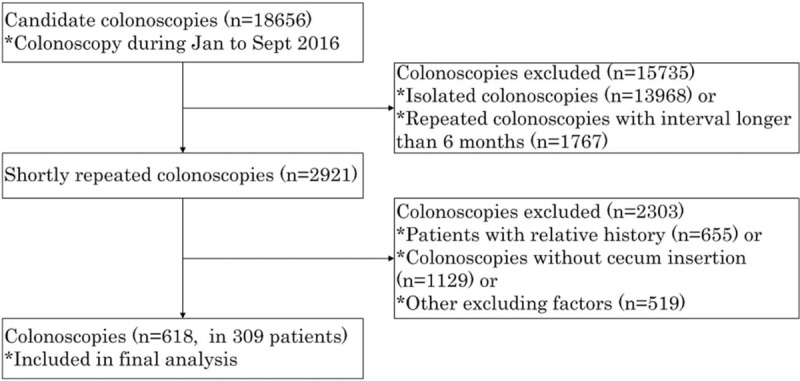
Flowchart of patients’ enrollment.

To collect repeated colonoscopies of same patients within limited time frame to minimize the possibility new adenomas emerging, we search the database for all the colonoscopies during January 2016 to September 2016. Patients were 18 years or older, and during this period they received total colonoscopies (cecum insertion) twice at least, of which the first one was not therapeutic. People who suffered from designated illness (polyposis, inflammatory bowel disease), received colectomy or received repeated colonoscopies with interval longer than 6 months were excluded. The inclusion process was shown in Figure 1.

### 2.3 Procedure

Patients were permitted to eat low-residue foods on the day before colonoscopy. We prescribed 2 L of polyethylene glycol (PEG) 4000 electrolyte powder (Wanhe, Shenzhen, China) or 50 g magnesium sulfate the night before the procedure. All colonoscopies were performed by experienced endoscopists, all of whom had at least 3 years’ experience in colonoscopy. We performed colonoscopy by using Olympus colonoscope (CF-Q260AI; Olympus, Tokyo, Japan). Patients were conscious during colonoscopy or received sedation by using intravenous propofol. Cecal insertion was achieved when ileocecal valve was seen directly. Bowel preparation quality was assessed as excellent, good, fair, poor or inadequate according to Aronchick scale.^[10]^ GI assistants helped with the procedure (patients position change, abdominal compression, polyp removal and other treatment) when necessary. Polyps removed were sent for pathologic exam.

### 2.4 Outcome measures and statistical analysis

According to former researches’ classification, lesions were grouped as diminutive (1–5 mm), medium (6–9 mm) and large (10 mm or larger).^[11]^ Histology details of adenoma includes tubular adenoma, villous adenoma, sessile serrated adenoma, and polyps with low- or high-grade intraepithelial neoplasia. Advanced adenomas are defined as adenomas larger than 1 cm, with villous component, high grade intraepithelial neoplasia or invasive cancer. Locations were grouped as cecum, ascending colon, transverse colon, descending colon, sigmoid colon, and rectum from the proximal to the distal. To explore the influence of location on miss rate, the total large bowel was divided by different points (hepatic flexure, splenic flexure, descending-sigmoid juncture, and recto-sigmoid juncture).

AMR is the main outcome, which refers to the proportion of adenomas missed during first colonoscopy to the total adenomas found during either colonoscopy.^[4]^ AMR is compared by different size, histology (advanced or not), bowel preparation quality, and location. Miss rate by patients is also calculated, which is defined as the proportion of subjects who has adenomas not detected in the first colonoscopies. Factors related to patients including age, gender, and bowel preparation quality are also compared. Adenoma detection rate is also calculated.

Categorical data are presented as number and percentage and compared by *χ*^2^ test. Continuous variables are expressed as median and ranges or mean ± standard deviation (SD), and compared with the Student's *t*-test. Variables that were predictive at the 0.05 level using univariate analysis were entered into the final multivariate analysis, and multivariate logistic regression was performed to identify risk factors for missing adenomas. Two-tailed *P*-values <.05 were considered statistically significant, and all statistical analysis of the data was performed with Microsoft Excel (Microsoft Corp., Redmond, WA), and SPSS Statistics 19 (IBM, Armonk, NY).

## 3 Results

### 3.1 1. Baseline characteristics

A total of 2921 repeat colonoscopies were recorded during research time frame. After excluding colonoscopies failed to insert cecum or of which patients had relevant history or other excluding factors, 618 colonoscopies (309 pairs) were retrieved. No severe complications happened. The median age of these patients was 58.5 years (range 20–87), and 62.4% were males. Indications of the first colonoscopy included screening, surveillance and other reasons (such as abdominal pain, anemia, rectal bleeding, and so on), and the reasons for a shortly repeated colonoscopy included endoscopic polypectomy after a positive colonoscopy, surveillance after polypectomy, reexamination after a colonoscopy of inadequate bowel preparation, re-examination by a senior clinician after a colonoscopy finding a colonic neoplasm and so on. Bowel preparation quality was grouped as Good (Aronchick's Excellent and Good) and Poor (Aronchick's Fair and Poor). The characteristics of the entire patient population are shown in Table 1.

**Table 1:**
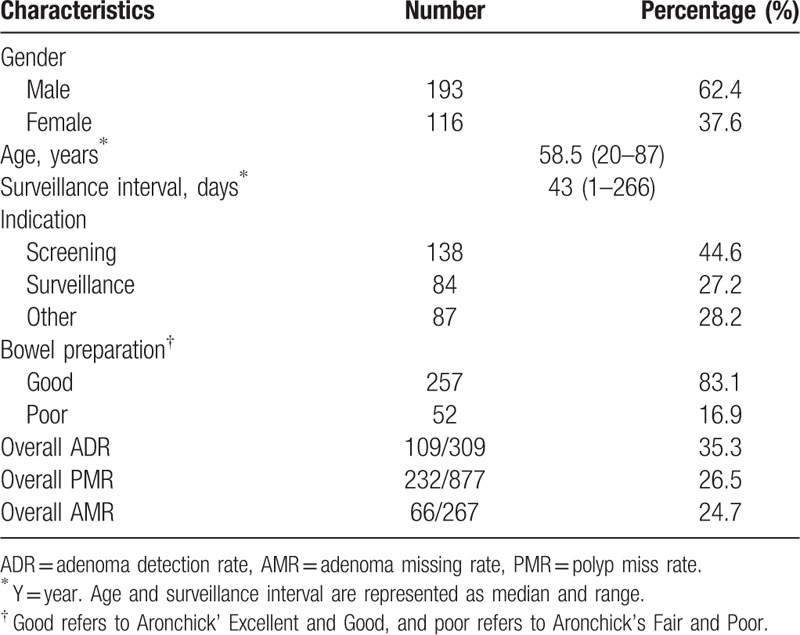
Baseline characteristics of patients (n = 309).

Notably, there were total 35 endoscopists involved in the analysis, and the endoscopists of the 1st and 2nd endoscopies were the same doctors in 71 patients (71/309, 23.0%).

### 3.2 2. Adenoma miss rate and its risk factors related to lesion and patient

There were totally 877 polyps detected in either colonoscopies, and 232 polyps were only detected in the second exams, so the overall polyp miss rate (PMR) is 26.5%. Around 267 adenomas were detected during the 618 colonoscopies, of which 201 adenomas in 109 patients were detected in the first colonoscopies and 66 adenomas were missing. So ADR was 35.3% (109/309), and AMR was 24.7% (66/267). Among the 267 adenomas, there were 120 diminutive, 71 medium and 76 large ones. Data of all the adenomas are shown in Table 2.

**Table 2:**
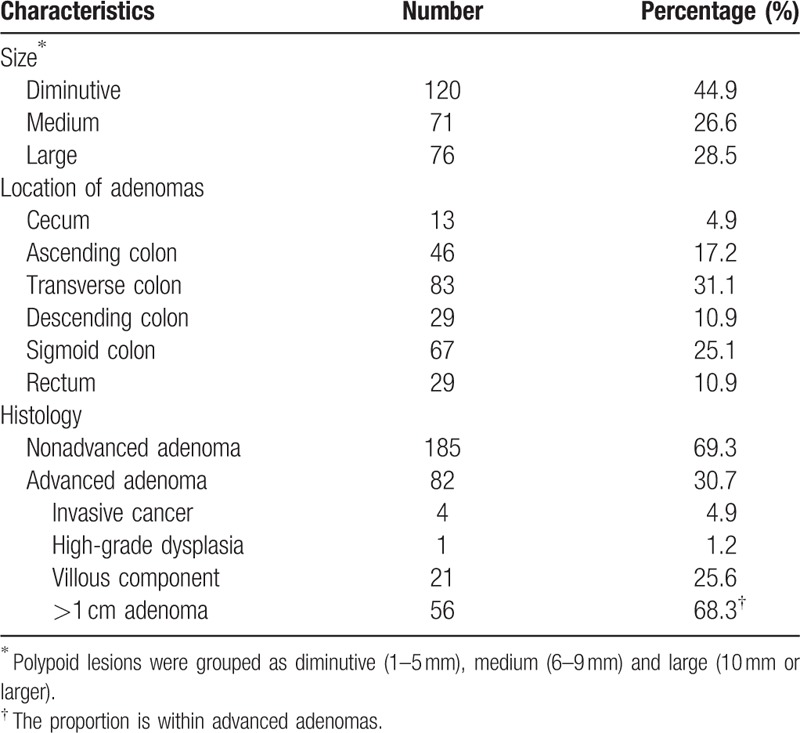
Baseline characteristics of adenomas (n = 267).

Risk factors related to missing adenoma between the detection and missing groups are summarized in Table 3. In univariate analysis, smaller in size (*P < *.001), nonadvanced (*P < *.001), poor bowel preparation (*P = *.028), and different location (*P = *.016) were associated with missing adenoma. In multivariate analysis, diminutive in size (odds ratio [OR] = 8.393, 95% confidence interval [CI] = 1.046–67.358, *P = *.045), poor bowel preparation (OR = 4.138, 95% CI = 1.837–9.324, *P = *.001) and located in ascending colon (OR = 6.628, 95% CI = 0.757–58.002, *P < *.001), transverse colon (OR = 6.271, 95% CI = 1.175–33.463, *P = *.032) or sigmoid colon (OR = 13.897, 95% CI = 2.520–76.632, *P = *.003) were also found to be independently associated with missing adenoma. Furthermore, by different dividing points, we found AMR was significantly high in cecum and ascending colon (*P = *.028) and low in rectum (*P = *.018).

**Table 3:**
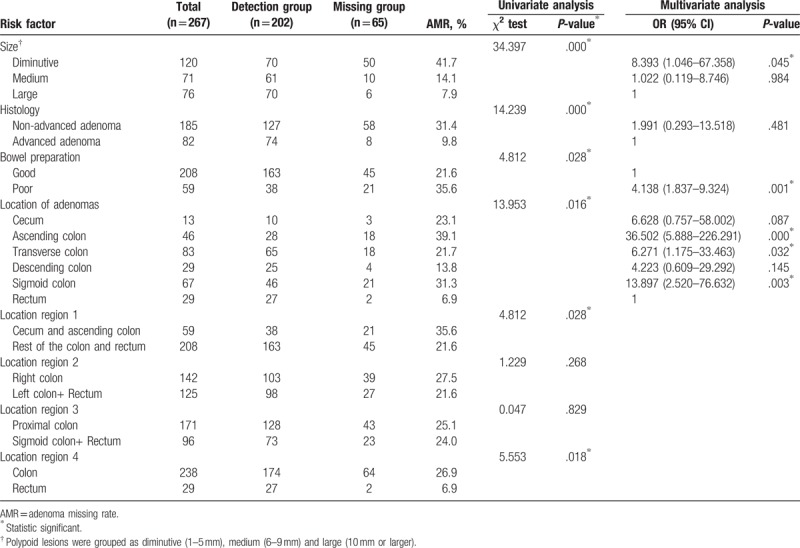
Risk factors related to missing adenomas.

As to risk factors related to patient, analyses are summarized in Table 4. In univariate analysis, ≥60 years (*P = *.006), male sex (*P = *.014) and poor bowel preparation (*P = *.006) were associated with missing adenoma. In multivariate analysis, all the 3 variables, ≥60 years (OR = 2.646, 95% CI = 1.114–6.289, *P = *.027), male sex (OR = 2.652, 95% CI = 1.019–6.903, *P = *.046) and poor bowel preparation (OR = 3.203, 95% CI = 1.183–8.674, *P = *.022), were also independently associated with missing adenoma.

**Table 4:**
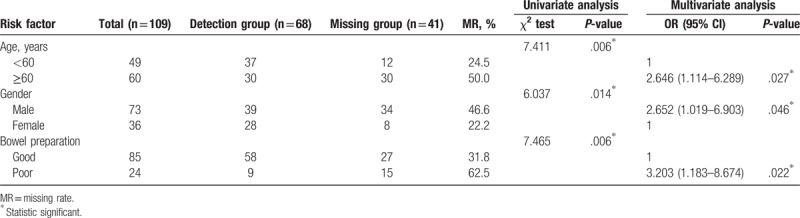
Risk factors related to patients with missing adenoma.

## 4 Discussion

The only sure way to reduce cancer is through screening and healthy lifestyle.^[12]^ CRC is one of the very cancers which are suitable for screening, and the colonoscopy-led screening program has been shown to reduce colorectal cancer incidence and mortality in the United States and other developed countries. But organized screening programs are still to be implemented in most countries.^[13]^ In China, CRC screening is still at the early stage, and community-based screening programs are in progress in some places like Shanghai, Tianjin, and Guangzhou. Questionnaire survey and fecal occult blood test are the primary methods rather than colonoscopy due to the short of medical resources and the poor compliance of colonoscopy, and screenees with positive results of primary test will be sent to endoscopy centers for further examinations. Chinese researchers are working on developing a product of stool DNA test of methylated syndecan-2 for colorectal neoplasia screening, and the application prospect is promising.^[14]^

The utility of colonoscopy and endoscopic polypectomy in CRC prevention is highly recommended, and colonoscopy is regarded as the golden criteria in CRC screening.^[15]^ While the truth is adenomas are commonly missed during colonoscopy with a poor AMR of 22%.^[8]^ Though highly recommended, colonoscopy is not a perfect method in adenoma detection. Actually, many endoscopists have missed many polyps during colonoscopy, and here we report an overall AMR of 24.7%. So it is an important issue to improve the quality of colonoscopy and not to ignore adenomas during colonoscopy before a better method which can take place of it comes out. Quality control is a big issue in colonoscopy research. ADR as the core index in quality control, which has been proven to be directly related to interval CRC, has already been recommended to be used in assessment of endoscopy centers and endoscopists by several national and societies’ guidelines. For example, ASGE guideline has set an ADR of 25% and 15% for male and female American white screenees older than 60 year old as the minimum standard in 2006,^[16]^ and the benchmarks have been updated to 30% for men and 20% for women in 2015.^[17]^

However, ADR does work, but is not enough for quality control. Wang *et al.*^[18]^ has proven that ADR is necessary but insufficient for distinguishing high versus low endoscopist performance, and a endoscopist of high ADR can still miss many adenomas in patients who already have adenomas detected. We now know that not only identifying a patient with adenomas is important, but also identifying each single adenoma is equally important, because any missing adenoma is a potential CRC in the future, leave out it is within a patient with or without adenomas detected.

AMR pays attention to every single adenoma instead of person suffering from adenomas, so it can be more sensitive than ADR. However, to calculate AMR, tandem colonoscopies must be taken, which cannot be routinely performed in daily practice, and previous AMR data are almost from clinical trials. The limitation of AMR utility makes it uncommon in colonoscopy quality control and its importance and superiority are not well accepted by endoscopists. Nevertheless, the endeavor to calculate AMR using retrospective data has been made. Bensen et al^[19]^ retrieved 76 pairs of colonoscopies performed within 4 months from approximate 15000 examinations to calculate AMR, and found an AMR of 12.0%. They also indicated that tandem colonoscopy or repeated colonoscopy could be used to calculate the true 1-yr recurrence of colorectal adenoma when incorporated with 1-yr re-examination. Kasugai et al^[20]^ retrieved the information of 688 patients who accepted repeated colonoscopies within 1 month, and discovered an AMR of 13.9%. Recently, Shin et al^[21]^ analyzed data of referral patients with advanced adenomas to be resected in the referring hospital, and a per-patient miss rate of adenoma of 47.2% was documented. These work shows AMR from retrospective data is theoretically practicable and comparable to that from tandem exams.

In our study, the overall AMR is 24.7% and the AMR of the cecum and ascending colon is 35.6%, and the data indicates proximal adenomas are prone to be missed. Soetikno et al^[22]^ have indicated that adenomas in proximal colon are more often flat or depressed and are therefore harder to be detected than pedunculated ones in distal colon. Moreover, in Pickhardt et al's^[23]^ study of location of missed adenomas, 14 (93.3%) of 15 nonrectal missed adenomas were located on a fold and 10 (71.4%) of these were located on the backside of a fold. When the colonoscope comes into proximal colon, difficulty of turning around and looking back makes the problems above emerge. It is well-reasoned that AMR of proximal adenomas is higher than that of distal ones. Our data also support the viewpoint. Other risk factors such as small in size, nonadvanced in histology, poor bowel preparation, old age and male are also associated with adenoma missing, and the attempts to reduce AMR may follow these cues.

Table 5 shows some previous data of AMR, and they vary widely from each other. The results of tandem exams should be compared with cautions, and let alone that of repeated ones. Factors including patients’ age, indications, bowel preparation, endoscopists, and instruments all impact the results. Unlike screening population are set as the standard target for ADR calculation, there is not a standard population for AMR, which makes the results various. Here we put forward data of missing adenomas in context of routine clinical practice in China, and we think many factors of the population enrolled influenced the comparability of the results, just like what matters in ADR.^[24]^

**Table 5:**
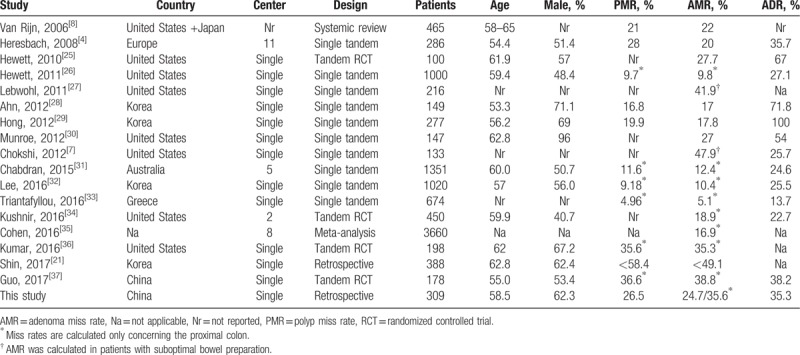
Previously reported AMR.

The inherent drawbacks of retrospective analysis cannot be avoided. Confined data resource only provide us limited factors for analysis, also with compromised data quality. We are unable to put forward factor analysis other than size, location and bowel preparation quality. We routinely use PEG4000 or magnesium sulfate for bowel preparation, but the retrospective data failed to provide us the difference on AMR between these 2 regimens, because the information was not always recorded completely. However, the difference might exist when these 2 regimens brings different bowel preparation quality. Procedure time has been proven to influence ADR due to doctors’ fatigue, and it may also influence AMR. But the difference exists in afternoon colonoscopies performed in full-day blocks by same endoscopists when ADR is analyzed, we herein cannot do this analysis based on the retrospective data. The same situation happens to the influence of withdrawal time on AMR, which we believe must be solved by randomized controlled trials. The proper of sample size is worth discussing. Longer enrollment timeframe provides larger sample size, but brings the possibility of adenoma emerging rather than missing. Our research is a preliminary one, and further prospective studies concerning the risk factors will surely bring us more valuable information, how to decrease AMR in the proximal colon, for example.

## Acknowledgments

We thank the subjects for their participation. We appreciate very much for all the endoscopists and pathologists in Changhai Hospital for their hard work. The last but not the least, we appreciate Dr Kai GU and Dr Yangming GONG (Shanghai CDC), Dr Jiaxin LI (Tianjin Nankai Hospital), and Dr Yufeng Chen (The Sixth Affiliated Hospital, Sun Yat-sen University) for sharing the information about CRC screening in their corresponding fields.

## Author contributions

**Conceptualization:** Zi-Ye Zhao.

**Data curation:** Cheng-Long Wang, Zhi-Ping Huang, Kai Chen.

**Formal analysis:** Cheng-Long Wang, Zhi-Ping Huang, Kai Chen.

**Funding acquisition:** En-Da Yu.

**Investigation:** Cheng-Long Wang, Zhi-Ping Huang, Kai Chen, Fei-Hu Yan, Liang-Liang Zhu, Yong-Qi Shan, Yong-Jun-Yi Gao, Bai-Rong LI.

**Methodology:** Zi-Ye Zhao.

**Project administration:** En-Da Yu.

**Resources:** Fei-Hu Yan, Liang-Liang Zhu, Yong-Qi Shan, Yong-Jun-Yi Gao, Bai-Rong LI.

**Software:** Zi-Ye Zhao.

**Supervision:** Hao Wang, En-Da Yu.

**Validation:** Hao Wang, Zi-Ye Zhao.

**Visualization:** Zi-Ye Zhao.

**Writing – original draft:** Zi-Ye Zhao.

**Writing – review & editing:** En-Da Yu, Zi-Ye Zhao.

Zi-Ye Zhao orcid: 0000-0002-0243-2873
